# Geodetic Network Design and Optimization on the Active Tuzla Fault (Izmir, Turkey) for Disaster Management

**DOI:** 10.3390/s8084742

**Published:** 2008-08-19

**Authors:** Kerem Halicioglu, Haluk Ozener

**Affiliations:** 1 Bogazici University, Kandilli Observatory and Earthquake Research Institute, Geodesy Department, Cengelkoy, 34680, Istanbul, Turkey; E-mail: kerem.halicioglu@boun.edu.tr; 2 Istanbul Technical University, Department of Geodesy and Photogrammetry Engineering, Surveying Technique Division, Maslak, 34469, Istanbul, Turkey

**Keywords:** Crustal Deformation, Tuzla Fault, Network Design and Optimization, Seismic Hazard, GPS sensors

## Abstract

Both seismological and geodynamic research emphasize that the Aegean Region, which comprises the Hellenic Arc, the Greek mainland and Western Turkey is the most seismically active region in Western Eurasia. The convergence of the Eurasian and African lithospheric plates forces a westward motion on the Anatolian plate relative to the Eurasian one. Western Anatolia is a valuable laboratory for Earth Science research because of its complex geological structure. Izmir is a large city in Turkey with a population of about 2.5 million that is at great risk from big earthquakes. Unfortunately, previous geodynamics studies performed in this region are insufficient or cover large areas instead of specific faults. The Tuzla Fault, which is aligned trending NE–SW between the town of Menderes and Cape Doganbey, is an important fault in terms of seismic activity and its proximity to the city of Izmir. This study aims to perform a large scale investigation focusing on the Tuzla Fault and its vicinity for better understanding of the region's tectonics. In order to investigate the crustal deformation along the Tuzla Fault and Izmir Bay, a geodetic network has been designed and optimizations were performed. This paper suggests a schedule for a crustal deformation monitoring study which includes research on the tectonics of the region, network design and optimization strategies, theory and practice of processing. The study is also open for extension in terms of monitoring different types of fault characteristics. A one-dimensional fault model with two parameters – standard strike-slip model of dislocation theory in an elastic half-space – is formulated in order to determine which sites are suitable for the campaign based geodetic GPS measurements. Geodetic results can be used as a background data for disaster management systems.

## 1. Introduction

This study suggests a plan for a large scale crustal deformation monitoring project including the relations between the global tectonics, the interpretation of seismicity and tectonics of the study area, appropriate geodetic techniques for deformation monitoring, combination of different techniques, geodetic network design and optimization. Deformation measurements performed using geodetic techniques include some critical steps in the processing and design stages. Moreover, other parameters such as the location of deformed area or the deformation type should also be taken into consideration. An appropriate technique should be chosen considering the deformation type, the proximity of the deforming area or object to urban areas and suitable processing techniques.

**Figure 1.**
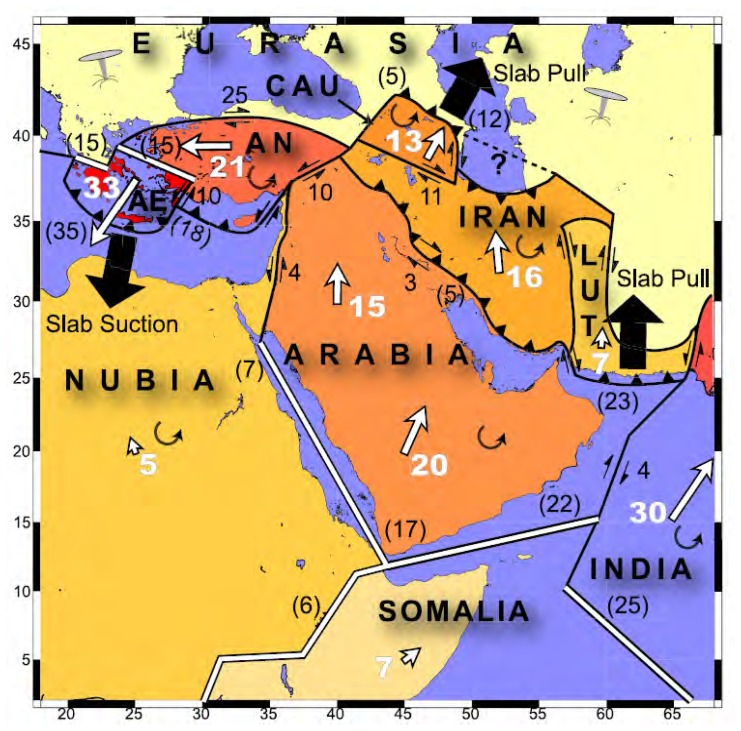
Plate interactions of Arabia-Africa-Eurasia zone [1].

The Aegean Region and Western Anatolia are one of the most seismically active and deforming parts of the Alpine-Himalayan orogenic belt. Consequently high seismic activity has been observed in this region. An extensional deformation regime has led to subsidence of the continental crust over all regions behind the south Aegean. The region is mainly under pure shear stress from an internally deforming counter-clockwise rotation of the Anatolian Plate relative to the Eurasian one. There is a multi disciplinary research report in the literature concerning the plate interactions through the whole Arabia-Africa and Eurasian plates performed for several periods [1]. Figure 1 shows the result of this study, performed by Reilinger *et al.* The Aegean Region has been suffering active N-S extensional tectonics, under the control of two main motions. One of the motions is the westward escape at a rate of 20-25 mm/yr of the Anatolian plate, bound by the North Anatolian Fault and East Anatolian Fault, and intersecting at the Karliova depression of the East Anatolia. The westward motions change direction in West Anatolia with a rather abrupt counter-clockwise rotation towards the southwest over the Hellenic Trench. The other motion is the N-S extension of the Western Anatolian and the Aegean plates with a rate of about 3-6 cm/yr. As a result of these motions a group of E-W trending grabens have been developing. These grabens are bound by E-W trending normal fault zones which extend about 100-150 km. These fault zones are generally segmented and each segment is no longer than 8-10 km [2].

The complicated geology of the region has given rise to disagreements on the source or beginning of the extension of the region. McKenzie [3] suggests the beginning time of the extension as 5 Ma, while by other researchers have suggested 13-11 Ma [4]. This variety in the suggestions concerning the beginning of the N-S extension for the Aegean Region may be due to on the insufficient accuracy of the methods used to determine the beginning time or lack of information about the previous geological researches that preclude accurate estimations.

Consequently, the result has been a focus of the geological investigations on the Aegean Region in order to understand the tectonics of the area. Geodesy and geodynamics can also contribute additional information [5]. Geodesy builds its investigations on the information gathered from the seismological studies. Therefore, interpretation of earthquake distributions, determination of focal mechanisms and field studies that aim to define fault traces provide valuable data for geodetic crustal deformation studies. Thus, the project area that is to be monitored with geodetic techniques has to be evaluated in terms of the project area's seismicity. A complete picture for deformation monitoring studies using geodetic techniques has to be formed including definitions on tectonics of the study area, network design regarding to the geological and geophysical parameters of the region and approaches to the combination of different geodetic techniques. The paper discusses the possible extensions in the size of the network depending on the fault characteristics. Monitoring two or more faults together, for instance, can be a better solution to understand the characteristics of the region in some cases so the study area needs to be extended during the geodetic observations. This paper uses the formulations in one-dimensional fault model with two parameters standard strike-slip model of dislocation theory in an elastic half-space for selecting suitable site locations of the network.

## 2. Micro-Tectonics Features of the Region of Interest

**Figure 2.**
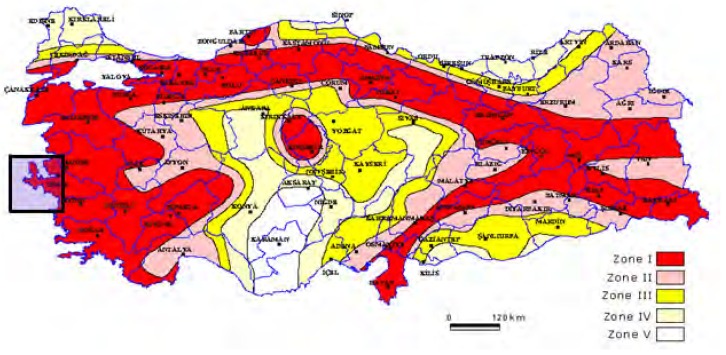
Turkey earthquake hazard map and study area [6].

The studied region has a high seismic activity due to the extensional regime of the Aegean Region. Thus Western Anatolia contributes greatly to Turkey's earthquake activity and neotectonics. Ozmen *et al.* [6] produced a seismicity map considering the data beginning from the instrumental time to present that indicates the different perspective of western Anatolia than the Turkey's total activity. Figure 2 shows the seismic risk zones and the study area which is in the high risk-zone I.

There are two main seismic belts within the boundaries of the region. One of them lies in the Crete-Rhodes-Fethiye and Burdur direction and the other one is in a direction along the Simav-Emet-Gediz and Afyon locations. These two belts have the highest seismicity in the whole Aegean Region [7, 8]. Geodynamic studies show that the Aegean Region needs to be investigated continuously with different scientific techniques. This study is going to subject the geodetic contribution to regional tectonics with some geodetic optimization techniques using gathered information from different sources. Although the tectonics of Eastern Mediterranean have been explained by long term episodic and continuous GPS observations [1, 9] some special cases need to be defined in specific regional deformations. Izmir as a high populated city settled on seismically active faults. Thus, there is always high seismic risk underlined in many studies [10-15] in Izmir, like the North Anatolian Fault Zone.

Several GPS network optimization studies have also been published during the last decade [16-18]. Therefore, there is a need to perform a large scale crustal deformation monitoring study using the results of previous studies mentioned above, in order to evaluate regional tectonics. However, the tectonics of Izmir and its vicinity is very complex in the geological sense and should be investigated in detail to understand long and short term geodynamic activities.

The deformation pattern in the Mediterranean region which forms a low elevated part of the Alpine Himalayan belt is rather complex, and usually occurs in the continental collision zones. The Aegean region is bounded to the north by the stable continental Eurasian plate, to the west by the Adriatic region, to the east by the central Anatolian plate, and to the south by the oceanic material beneath the Mediterranean Sea, which is northern edge of the African plate. The Black and Mediterranean Sea floors have mean depths of 1500 and 1300 meters, respectively, while the Aegean Sea floor has a mean depth of 350 m. In other words, the Aegean Sea floor may be seen as a high plateau between the deeper Black Sea and Mediterranean Sea floors. The Aegean is characterized by a relatively thicker crust (25-30 km) than a typical oceanic crust, which might conversely be interpreted as a thinned continental crust. The Aegean is also situated in the convergent boundary between the African plate and Eurasian plate. The African plate has rotated counter-clockwise with respect to Eurasian plate during the last 92 Ma [19]. The spatial distribution of earthquakes and detailed topographic studies indicate the existence of a northward-dipping subducted slab beneath this region (African plate beneath Eurasian plate). However, according to Müller *et al.* [19], a roughly N-S directed lithosphere shortening rate is increasing from west to east in the Aegean region. The region is also characterized by high heat flow, which is related to thin and deformed (stretched) continental crust. This thinning is continuing until now and for this reason, it is the worldwide most seismically active and internally deforming area of the entire Alpine-Himalayan belt and at of all continents [4, 20].

Papazachos [21] defines the northern and eastern boundaries of the Aegean plates which comprises the Hellenic Arc, Greek mainland and western Turkey. The Anatolian plate has a relative motion of 22-25 mm/yr with respect to the Eurasian Plate according to McClusky *et al.* [22]. The focal mechanism solutions of earthquakes indicate that the faulting in the western part of the Aegean region is mostly extensional in nature on normal faults, with a NW to WNW strike and slip vectors directed NW to N [23]. The evidences from paleomagnetism show that this region rotates clockwise relative to a stable Eurasia. According to Piper *et al.* [24], paleomagnetic data in the eastern Aegean Region is consistent with very small or no rotations in the northern part and possibly counter-clockwise rotations in the south relative to the Europe, including some ambiguities. The strike-slip faulting that lying through the central Aegean from the east appears to end abruptly in the SW against the NW trending normal faults of Greece.

**Figure 3.**
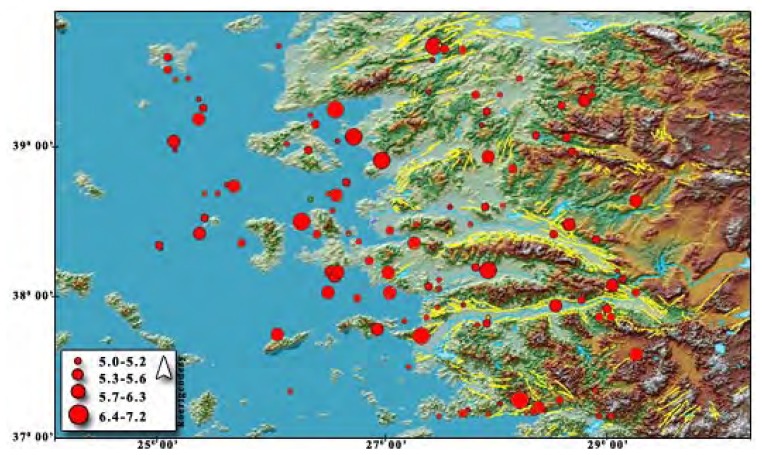
Faults and earthquakes, M>5 in 1900-2006 [8].

**Figure 4.**
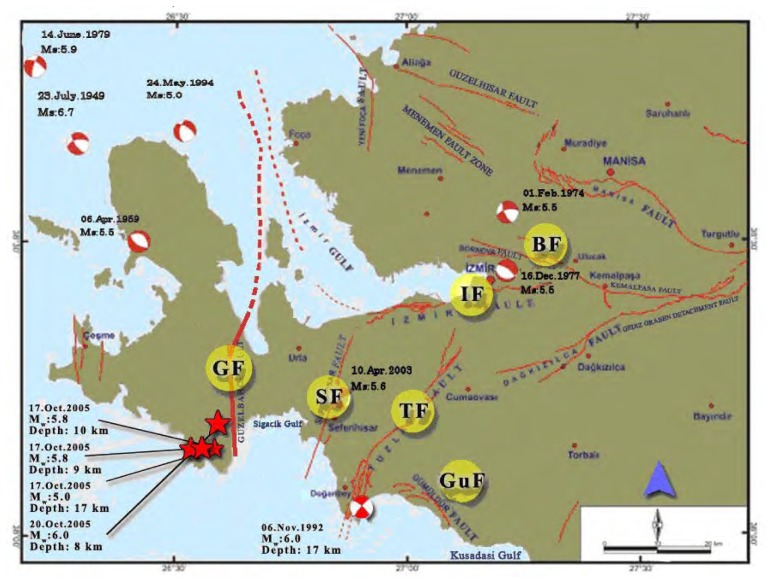
Important faults of Izmir and its vicinity modified from [25] (GF: Guzelhisar Fault, IF: Izmir Fault, BF: Bornova Fault, TF: Tuzla Fault, SF: Seferihisar Fault, GuF: Gumuldur Fault).

The extension tectonic regime affected Western Anatolia in the neotectonic age. Izmir lies on the west side of the Gediz Graben and bound by the Gulf of Izmir. There are several active faults that have triggered the dense earthquake activity recorded beginning from the 20th century as shown in Figure 3. In addition some major faults have the capacity to produce big earthquakes. According to the report on Active Faults and Seismicity in Izmir and its vicinity [25], there is not enough investigation on the earthquake activity potential except for the Gediz Graben. The report defines active faults within a 50 km semi-diameter area which has an origin at central Izmir. Emre *et al.* [25] defined the 14 active faults shown in Figure 4 through the region. These faults are Guzelhisar, Menemen, Yenifoca, Izmir, Bornova, Tuzla, Seferihisar, Gulbahce, Gumuldur, Gediz Graben detachment faults, Daglikizca, Kemalpasa, and Manisa Faults. The following paragraphs give brief explanations about these active faults and focus on the Tuzla Fault in detail.

Tuzla fault is in the southwest of Izmir, between Cape Doganbey and Gaziemir counties with an alignment trending NE-SW. It has been known by different names in the literature, such as Cumaovasi and Orhanli faults [26, 27]. The fault is 42 km long through the land side. However, in 2004 and 2005, after the investigations performed by GDMRE Sismik-1 research ship in Cape Doganbey the total length published was more than 50 km. Tuzla fault has three main segments that have different directions. Emre et al. [25], named these three parts the Catalca, Orhanli and Cumali sections, arranged from north to south, respectively. Therefore, the right-lateral strike slip Tuzla Fault, with its 50 km length (including the undersea segments) is considered as an active and important tectonic phenomenon of Izmir and its vicinity. On the other hand, Tuzla Fault is the main element that defines the paleo-geography of the region during the Miocene period [28]. Genc *et al.* also claimed that the fault has left-lateral strike slip behavior. To the contrary, some other studies [22, 25, 26] propose that the fault had a right-lateral strike slip behavior during the Quaternary. The fault plane solutions determined by Turkelli *et al.* [29, 30] also confirm this theory. Important earthquakes within last two decades which confirms the seismic risk of the Aegean Region are shown in Table 1.

## 3. Design and Implementation

Observation techniques, selected equipment and surveying interval of any project have to be optimized in terms of several parameters. These optimizations, in general, are realized to achieve a desired precision. Besides, reliability is also as important as precision. One should trust not only the results but also the reliability of a network which can be expressed as mathematical relations. The precision, reliability and economical parameters in a geodetic network can be arranged in order to achieve the optimum solution which is defined as the optimization of geodetic networks [31]. In order to determine the deformation, generally local networks are preferred. A deforming area is generally covered by a number of control points. These points constitute a geodetic network and their location or structure is defined by the topographic and geological parameters. The number of points is directly related with the deforming object and the deformation accepted in the area. The ideal approach is an interdisciplinary study to define the number of points and locations for these “control networks”. Not only geodesy but also geology, geophysics and disaster management should contribute deformation monitoring studies. Ayan [31] has suggested three sets of control points for deformation monitoring which are deformation points, reference points and orientation points.

In order to contribute geodynamic studies, after the 2005 M: 5.9 Sigacik earthquake, this region's seismic risk was considered. Izmir has a very complex geological structure of faults with different characteristics. This variety in fault characteristics also made it difficult to select the project area. For this reason, at the beginning the information collected about the region covered the whole Western Anatolia region, but then the research had focused on the most important section of the region. Tuzla Fault and its vicinity coincide with the aims defined at the beginning of the study because of the active behavior of the fault, its closeness to Izmir and big earthquakes recorded in the area.

Geodetic deformation analysis requires a stable, continuously or periodically observed network. Moreover, in order to estimate the small amount of deformations, some additional techniques such as precise leveling and gravimetric or astrogeodetic techniques are generally considered. Leveling routes are generally designed in perpendicular lines with respect to the fault trace. This paper, which focused on the Tuzla fault, designed a micro-geodetic network considering the valuable information gathered from different resources such as municipalities and then state the theoretical background with some scientists' approaches in terms of geodetic optimization.

The general plan for the network design performed on several parameters which are the available data collected from local resources, the topographic and economic situations, equipment which is going to be used and the fault geometry. The outputs of these parameters are the approximate locations of the geodetic control points, the number of the stations, and the observation and processing strategies.

Especially over the last two decades studies in the area of crustal deformation along plate boundaries and individual fault traces have grown, so the interest in an optimal design of monitoring schemes has increased. Because of the effectiveness of GPS for crustal deformation monitoring processes, the optimal design of monitoring network becomes a great practical interest. Designing a geodetic network can be generally divided into four main stages. The Zero-Order Design (ZOD) which generally deals with the definition of the optimum reference system of the network. The First-Order Design (FOD) involves the geometric shape of the network including the optimum number and locations of the geodetic stations. The Second-Order Design (SOD) deals with the determination of the weights of network measurements. SOD interested in which observations and with what precision should be achieved in the network. Finally the Third-order Design (TOD) considers the improvement of an existing network including the additional measurements that has to be made with the desired precision and what weights are selected for the improvement of network. Schmitt [32] claimed that in cases where the period of time between consecutive observations is taken into account, the term Fourth-Order Design maybe used.

In order to define the number of station that should be added into a deformation network or which sites should be used for that purpose is directly concerned with the phenomenon understanding fault mechanics. Gerasimenko *et al.* [17] conceived a model for this purpose using a simple strike-slip fault model in which the deformations are parallel to the fault trace, in order to facilitate the solution. A one-dimensional fault model with two parameters standard strike-slip model of dislocation theory in an elastic half-space can be formulated as:

$$d(x)=-\frac{V}{\pi }\arctan\left(\frac{x}{H}\right)$$

where x is the distance perpendicular to the fault, and the fault plane extends from the surface of the half space to infinite depth, locked from the surface to H km, and freely slipping below this depth V millimeter per year. The method suggested by Blewitt [16] leads to exact analytical solutions for the ideal transform fault locked down to depth D. According to this method, to resolve the depth of locking D and the location of the fault simultaneously, optimal station locations are at *D/√3* from the *a priori* fault plane. The seismogenic zone which is obtained as 12 km, derived from earthquake depths using the information taken from KOERI earthquake catalogs [8]. In other words, geodetic sites which are chosen and established are around 7 km away from the fault trace. On the other hand, analysis of slip partitioning in two-fault system shows that the resolution is optimized by including a station between faults. If the distance between faults is greater than 2D which is approximately 30 km the resolution is limited. Design is also suitable for precise leveling on short baselines of the network in order to increase the vertical component accuracy of position by using precise leveling technique.

According to the optimization strategies, performed experiments and collected information stated above, a geodetic network has been designed in order to monitor Tuzla fault and its vicinity and interpretation strategies are discussed.

The network was designed based on the information from existing control points and the fault trace geometry. Some additional stations were established in order to define the locking depth and slip rate of the fault trace according to the conclusions defined above. Moreover, because of the possibility of the extension of the study area, other active faults were taken into consideration in the design process. The station names are identified using four character Turkish National Fundamental GPS Network (TNFGN) station names.

After discussions with the local administrations, 14 control stations were selected for the network from among hundreds of possible sites. Numerous station points have been established throughout the region, especially in last three years for cadastre projects.

**Figure 5.**
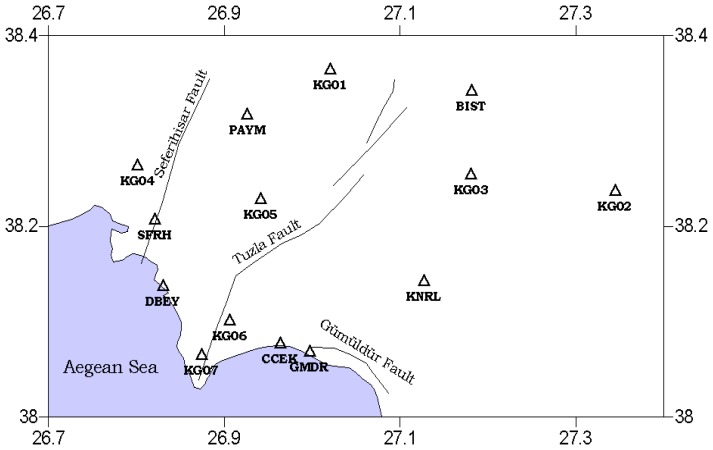
Locations of the sites of Izmir microgeodetic network.

Figure 5 shows the locations of the sites and an approximate trace of Tuzla and Seferihisar faults. Stations are distributed both on the fault trace and some 20 km away from the fault. The stations are close to each other along the south segment of Tuzla fault because the fault has a very complex and sectional structure in that area. This complexity, named the Cumali segment, is a zone of several faults that are parallel to each other [25]. The length of this segment is 15 km and it has 10 km long undersea part [15]. Moreover, this segment is very close to a 15 km long normal fault, Gumuldur fault, so this complexity has to be considered in any design process. Adding extra control points to the network would be a solution for monitoring this dynamic region of study area. There are some short baselines in the network such as CCEK-GMDR baseline because of the adjacency of two active faults. There is another fault very near to Tuzla fault and GMDR and CCEK points are very close to that Gumuldur fault. The WGS84 coordinates of the control station shown in Table 2.

In summary, the locations of the station points of the microgeodetic network are distributed on both sides of the fault. Moreover, some stations are located very near to the fault trace and some others as far as 20 km away from the fault trace, according to the distribution of the surface deformation with respect to the distance from the fault trace.

The network is compatible with the studies performed in first order network design studies. Generally the lines connecting GPS stations are in alignment with the direction of extension or compression, the angles of triangles composed by GPS stations are generally between 30 and 130 degrees [18]. On the other hand, some additional points that were added to the network like GMDR, KG07, KG06, and KG02 do not satisfy the above rules. However, those points were selected deliberately because of the very complex structure of the southern segment of the faults, composed of several pieces. KG02 was selected because we desired to evaluate the results in terms of short and long baselines and for various perpendicular distances to the fault trace.

Moreover, a block exists in the middle of Karaburun peninsula that has a differential motion at a rate of 3−5±1 mm/year to the east and 5−6±1 mm/year to the south [13]. Therefore, 14 points were thought to be enough for determining the slip rate, which is not as small, as stated by Gerasimenko *et al.* [17]. The network designed to be suitable for future studies which have a possibility to enlarge the project area, so the suggestions mentioned in Blewitt [16] are taken into consideration. The sites were also selected according to the transportation possibilities and visibility of open sky. The reconnaissance performed in the region made it easy to define those site properties.

## 4. Results and Discussion

This study focused on the idea of dealing with a crustal deformation monitoring project on a particular fault which has a high-seismic risk using geodetic techniques. Moreover, this study attempted to establish interactions between geosciences and geodesy in terms of deformation monitoring projects. The paper explains the tectonics of the eastern Mediterranean and Aegean Region in general and the tectonics of Izmir and its surrounding area in more detail. Important faults are underlined from a recent study performed by General Directorate of Mineral Research and Exploration in 2005 [25]. Some projects that have geodetic components were also investigated [1, 9, 13, 22] to focus on the movements of Anatolia and western Turkey.

**Figure 6.**
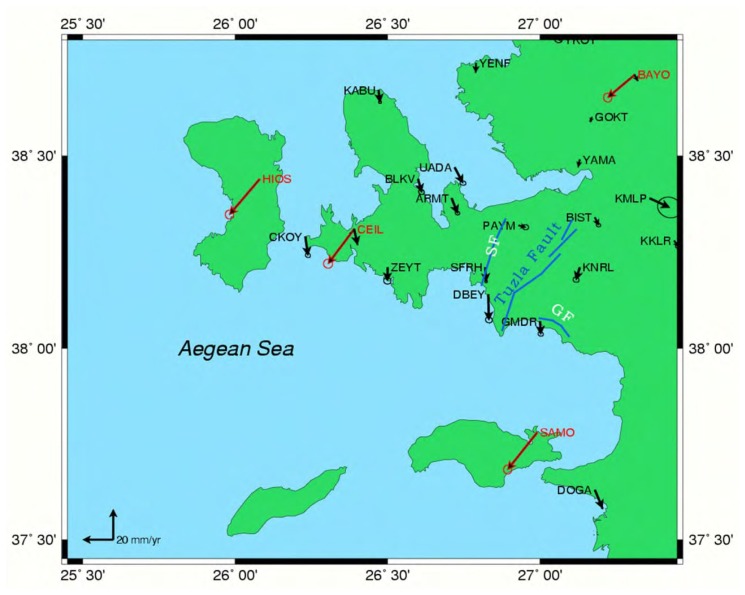
Red colored arrows indicate the Eurasia fixed velocity vectors, black arrows indicate the Anatolia fixed velocities [13, 22], blue lines indicate faults

According to the study of McClusky *et al.* [22], the high rate of velocity vectors especially in the Aegean Region is pointed out. Moreover, Reilinger *et al.* [1], mentioned the high rate of movement of western Anatolia according to the Anatolian plate. Another recent study [13] that covers an area between latitudes 37^°^ 45′ and 39^°^ 00′, and longitudes 26^°^ 00′ and 28^°^ 00′ mention the high rate of velocities especially near Tuzla fault. The velocities from two different studies can be seen in the Figure 6, where the black arrows indicates the residual velocities obtained by differentiating ITRF2000 and Eurasia plate velocities by using the following formula. On the other hand, red colored arrows indicate the Eurasia fixed velocity vectors. Figure 6 indicates an important deformation rate especially around Tuzla Fault.

$$v_{r}={\hat{v}}_{\text{ITRF}2000}-{\hat{v}}_{\text{PLATE}}$$

In order to contribute these projects by performing large scale fault based deformation monitoring study, Tuzla fault and its vicinity was selected considering its high seismic risk. Therefore, a reconnaissance was planned after the literature research in order to investigate the field and collect necessary information from local resources. Thus, this reconnaissance to the region was performed, the information collected, evaluated and analyzed within this study. Moreover, first order network design problems are quoted to create a harmony between microgeodetic networks. Network stations are selected from a large set of control points according to the suggestions mentioned in several studies. These whole processes produced a microgeodetic network that is selected from a huge set of information.

The network has an open end for future studies. In other words, there is a possibility of an extension for the network in order to monitor some additional faults. Tuzla fault exists in the center of the region and is very near to the big metropolitan city, Izmir. Thus the origin of the study is selected near this fault. Some researchers also mention the high seismic risk of the region including Tuzla fault [14, 15]. On the other hand, it is certain that, the area should be monitored by a larger and dense network with continuously operating GPS stations. For further studies, campaign based GPS observations are planned beginning from the current network designed in this study and will extend to the west to the Karaburun Peninsula, and to the east to the eastern Aegean region. According to the results achieved from some researches [14, 34, 35], there is a great seismic risk through the transform faults to the east near Pamukkale-Denizli. However, in this study, because of the topography related effects such as high mountains and the small rate vertical deformation make it nearly impossible to study with GPS or precise leveling techniques. For the reasons mentioned above, the network established to the area that is covering the Tuzla fault.

In conclusion, this paper, a plan for deformation monitoring studies using geodetic techniques including network design and optimization was prepared. The next step of this study will be three GPS campaigns on the designed network in two periods. In addition to GPS technique, conventional geodetic techniques such as precise leveling technique would be a choice for normal faults where small vertical deformations need to be determined. Further studies will be built on the information and techniques introduced in this study. It is certain that geodetic techniques are capable of determining small movements which are quite valuable information for earth sciences. Moreover, geodetic results can be valuable information for management systems in terms of the decision making based on characteristics of the geological features of the study area.

## Figures and Tables

**Table 1.** Important earthquakes in the region within the last two decades.

**Day**	**Year**	**Lat.**	**Lon.**	**Depth**	**M**
06.Nov	1992	38.16	26.99	17	6.0
28.Jan	1994	38.69	27.49	5	5.2
24.May	1994	38.66	26.54	17	5.0
10.Apr	2003	38.26	26.83	16	5.6
17.Apr	2003	38.24	26.86	6	4.8
17.Oct	2005	38.15	26.54	10	5.8
17.Oct	2005	38.15	26.53	9	5.8
17.Oct	2005	38.15	26.58	17	5.0
20.Oct	2005	38.18	26.59	8	6.0

**Table 2.** Locations of network stations.

**Site name**	**φ Latitude (in Degrees)**	**λ Longitude (in Degrees)**
KG01	38.36416	27.02072
KG02	38.23637	27.34451
KG03	38.25372	27.18084
KG04	38.26320	26.80143
KG05	38.22815	26.94152
KG06	38.10099	26.90646
KG07	38.06433	26.87388
PAYM	38.31700	26.92600
KNRL	38.14244	27.12700
GMDR	38.06800	26.99700
DBEY	38.13700	26.83000
SFRH	38.20700	26.82100
BIST	38.34200	27.18100
CCEK	38.07659	26.96351
